# Mucosal Blood Group Antigen Expression Profiles and HIV Infections: A Study among Female Sex Workers in Kenya

**DOI:** 10.1371/journal.pone.0133049

**Published:** 2015-07-17

**Authors:** Nadia Musimbi Chanzu, Walter Mwanda, Julius Oyugi, Omu Anzala

**Affiliations:** 1 Institute of Tropical and Infectious Diseases, College of Health Sciences, University of Nairobi, Kenya; 2 Department of Medical Microbiology, College of Health Sciences, University of Nairobi, Kenya; 3 KAVI Institute of Clinical Research, College of Health Sciences, University of Nairobi, Kenya; Institute of Infection and Global Health, UNITED KINGDOM

## Abstract

**Background:**

The ABO blood group antigens are carbohydrate moieties expressed on human red blood cells however; these antigens can also be expressed on some other cells particularly the surface of epithelial cells and may be found in mucosal secretions. In many human populations 80% secrete ABO antigens (termed ‘secretors’) while 20% do not (termed ‘non-secretors’). Furthermore, there are disease conditions that are associated with secretor status.

**Objective:**

To investigate correlations between secretor status and HIV infection among female sex workers in Nairobi, Kenya.

**Methodology:**

This cross-sectional study recruited 280 female sex workers aged 18–65 years from the Pumwani Majengo cohort, Kenya. Blood typing was determined by serological techniques using monoclonal antibodies to the ABO blood group antigens. Secretor phenotyping was determined using anti-H specific lectins specific to salivary, vaginal and cervical blood group H antigen using the agglutination inhibition technique and correlated to individual HIV sero-status. Participants were additionally screened for *Bacterial vaginosis*, *Neisseria gonorrhoea* and *Trichomonas vaginalis*.

**Results:**

Out of the 280 participants, 212 (75.7%) were secretors and 68 (24.3%) were non-secretors. The incidence of all infections: HIV, Bacterial vaginosis, Neisseria gonorrhoea and Trichomonas vaginalis was higher among secretors compared to non-secretors. However, this difference was only statistically significant for HIV infection incidence rates: HIV infected secretors (83.7%) versus HIV un-infected secretors (71.8%) (p = 0.029) Based on ABO phenotype stratification, the incidence of HIV infection was higher among blood group A secretors (26/52 = 50%), in comparison to B (12/39 = 33.3%: p = 0.066), AB (3/9 = 33.3%: p = 0.355), and O secretors (36/112 = 32.1%: p = 0.028).

**Conclusion:**

This is the first report to document the variable expression of the ABH blood group antigens profiling secretor and non-secretor phenotypes in the female genital tract among a high-risk population in a Kenyan population. These findings suggest the non-secretor phenotype may confer a certain degree of protection against HIV infection.

## Introduction

Thirty years since the discovery of the Human Immunodeficiency Virus (HIV), there have been numerous advances made in the field of HIV prevention and management. HIV/AIDS has been transformed from a fatality into a chronic manageable condition. Despite these advances, the pandemic remains a global emergency. Sub-Saharan Africa has been hit the hardest, bearing an estimated two-thirds of the burden, with 1.6 million Kenyans living with HIV [1]. Kenya faces a generalized HIV/AIDS epidemic, with a national adult prevalence rate of 5.6% that continues to exert a devastating toll on all sectors of society. In Kenya, women display a higher prevalence of 6.9% against the 4.4% for men [2] and with continuing success in HIV prevention efforts, the focus is now on a comprehensive understanding of the rate, distribution and establishment of new infections. The Kenyan epidemic is primarily driven by sexual transmission, which accounts for more than 90% of new infections. Of these 77% are through heterosexual intercourse [3]. Key populations include sex workers and their clients who account for 14.1% of all new infections [4]. Indeed, this high-risk population comprise an important ‘core group’ through which they serve as a bridge of HIV infection into the general population. Therefore, intervention programs targeting female sex workers, incorporating effective efforts and measures addressing the mucosal transmission of infection could have a huge public health impact towards curbing the HIV/AIDS pandemic.

Studies carried out over the past decade on female sex workers in Kenya, have established HIV transmission is primarily influenced by the genital mucosal microenvironment [5–7]. However, the association between the tissular distribution of ABO blood group antigens—secretor status—and HIV transmission remains an untapped area of research. The secretor phenotype is conferred by the *Fucosyltransferase 2* (*FUT2*) gene [8] and based on expression of the *FUT2* gene, approximately 80% of the population are ‘secretors’ and 20% are ‘non-secretors’ [9]. In secretors, the products of the *FUT2* gene, result in the phenotypic expression of the ABO antigens, on epithelial cells located on mucosal surfaces and in bodily secretions. This raises a number of questions on what role these antigens play at the level of interactions with pathogenic organisms on mucosal surfaces, which are often the first portal of entry for microorganisms including HIV.

Pathogens are always evolving, however, they are also highly selective agents. Therefore for microorganisms that preferentially bind to carbohydrate moieties, host cell surface molecules such as the ABO antigens can reveal patterns of selection. In non-secretors lack of expression of the blood group antigens confers a degree of protection against infections as seen in Norovirus (NoV), a pathogen which preferentially binds to blood group antigens expressed on the surface of mucosal epithelia of the gastrointestinal tract[10, 11]. Similarly, the non-secretor phenotype confers a degree of protection against *Streptococcus pneumonia* [12] and *Campylobacter jejuni* [13] both of, which favourably bind to the H type 2 histo-blood group antigens to establish infection. It is postulated, the histo-blood group antigens act as anchors to these pathogens and therefore play an important role in host susceptibility. Inversely, there is evidence from studies that suggests the expression of blood group antigens in mucosal secretions may provide a mechanism of evasion, by reducing pathogen attachment [14]. For example, in secretors addition of a fucose by the *FUT2* enzyme masks the uropathogenic *Escherichia coli*, and as a result secretors are less prone to recurrent urinary infections compared to non-secretors [15]. However, to date there have been no studies to investigate any possible associations between secretor phenotypes and infections in Kenya. This study was designed to investigate correlations between secretor status and HIV infection among female sex workers in Kenya based on the hypothesis, ABH secretors are more susceptible to HIV infections.

## Materials and Methods

This was a cross-sectional study conducted from January—October, 2013 in Nairobi, Kenya.

### Ethics Statement

This study was approved by the Kenyatta National Hospital—University of Nairobi Ethics and Research Committee. Written informed consent was obtained based on a non-coercive approach from all study participants.

### Study participants

Based on a consecutive sampling approach, 280 female sex workers aged 18–65 years presenting to sex worker outreach program clinics for routine screening were recruited consecutively for this study. The study participants were enrolled in the well-established Pumwani Majengo cohort in Nairobi, Kenya. The sample size was calculated using a standard formula, Hayes and Bennett [16] to compare the proportion of individuals with the outcome of interest the secretor phenotype. This was using an estimated power of 80%, a significance level of 0.05, the two populations: secretors versus non-secretors. The calculation was based on the evaluation of the impact of secretor status on HIV susceptibility: 26.7% for the secretors and 17% for the non-secretors as previously described i.e. non-secretors are less susceptible to HIV [17].

### Interviews and Examination

The volunteers were interviewed concerning socio-demographic characteristics, medical history and sexual behavior and underwent general physical and detailed gynecologic examinations.

### Specimen Collection

A 3ml sample of venous blood was obtained using standard phlebotomy techniques for ABO blood grouping and HIV-1 screening. Vaginal and cervical samples were obtained by sterile cotton swabs placed in two separate tubes containing 0.5mL of sterile saline, both for determination of secretor and non-secretor phenotypes. Additional cervical samples were obtained for detection of Bacterial vaginosis antigen and for screening for Trichomonas vaginalis and Neisseria gonorrhoeae organisms as indicated. Data regarding the stage of menstrual cycle, concurrent use of contraceptives and sexual behaviour, was taken under consideration at time of sample collection. This study excluded women who were pregnant based on urine pregnancy testing on-site.

### Sample Processing

Analysis was performed at the KAVI-Institute of Clinical Research laboratories, University of Nairobi, Kenya.

#### Blood Grouping

ABO blood grouping was determined using commercial antisera kits: murine monoclonal anti-A, anti-B and anti-D antisera (Plasmatec Laboratory Products Ltd., Bridport, Dorset, UK) by the standard conventional haemagglutination technique.

#### Secretor Phenotyping

Secretor status was determined using anti-H Lectin (Ulex Europaeus) (Lorne Laboratories Ltd., Reading, Berkshire, UK) to vaginal and cervical H antigen by the agglutination inhibition technique.

#### Microbiological Methods

Serological assays were performed on samples as indicated to test for HIV-1 rapid testing (Vironostika), Bacterial Vaginosis by microscopy (Gram Stain), Neisseria gonorrhea by plate culture using chocolate agar and Trichomonas vaginalis by Wet mount.

### Statistical analysis

Mantel-Haenszel χ^2^ tests were performed to compare dichotomous or categorical factors, with odds ratios (ORs) used as measures of association. Frequency distribution of the dependent categorical variables was compared by the Chi-square test. The level of significance for all the analyses was set at a value of P = 0.05 at 95% Confidence Interval (CI). Multivariate logistic regression analysis was used to estimate the OR and confidence interval (CI) for the association between HIV infection and the secretor phenotype, adjusted for potential confounding variables. The presence of confounding was determined by comparing the ORs obtained from logistic regression models before and after addition of the covariates being evaluated. Stratified logistic regression analyses were performed to assess the relationship between HIV infection and the secretor phenotype with female sex workers; comparing HIV infected and HIV un-infected. The association between HIV-1 and secretor status was independent of several potentially confounding variables including, age, nationality, contraceptive use, and for *Bacterial Vaginosis*, *Neisseria gonorrhea* and *Trichomonas vaginalis* infections.

## Results

Secretor phenotypes were determined for 280 female sex workers in Nairobi, Kenya. All study cases were enrolled in the well-established Pumwani Majengo cohort and were recruited from three sex worker outreach program clinics, located in Kawangware, Majengo and the Central Business District, Nairobi. The mean age of the women screened was 36.1 years (SD, 9.3 years). Socio-demographic characteristics including age, nationality, duration in prostitution, contraceptive use, and number of children are as summarized in Table 1.

**Table 1:** Socio-demographics characteristics of female sex workers recruited from the Pumwani Majengo Sex Worker cohort in Nairobi, Kenya (n = 280).

POPULATION CHARACTERISTIC	NUMBER OF STUDY CASES (n)
**Age Range (Years)**	
18–29	77 (27.5%)
30–39	113 (40.4%)
40–49	62 (22.1%)
50+	28 (10.0%)
Mean Age	36.1 years (SD, 9.3 years)
**Nationality**	
Kenyan	249 (88.9%)
Tanzanian	7 (2.5%)
Congolese	1 (0.3%)
Ugandan	2 (0.7%)
Declined to Disclose	21 (7.5%)
**Duration in Prostitution**	
0–2 years	59 (21.1%)
3–5 years	90 (32.1%)
6–8 years	39 (13.9%)
9+ years	92 (32.9%)
Mean Duration in Prostitution	8.75 years (SD, 7.9 years)
**Contraceptive Use**	
Oral Contraceptive Pill	20 (7.1%)
Intrauterine Contraceptive Device	5 (1.8%)
Depo-Provera	39 (13.9%)
Tubal Ligation	4 (1.4%)
Male Condom	196 (70%)
Female Condom	1 (0.4%)
Diaphragm	2 (0.7%)
Other	13 (4.6%)
**Number of Children**	
0–2	207 (73.9%)
3–5	69 (24.6%)
6+	4 (1.4%)

### Secretor Status

Each study participant was screened for secretor status, which was based on blood group H antigen expression in mucosal samples, saliva, vagina and cervix. Out of the 280 participants, 212 (75.7%) of the participants were secretors and 68 (24.3%) were non-secretors. Secretor and non-secretor phenotypes were consistent in swabs obtained from all three sites, oral, vaginal and cervical as shown in Table 2.

**Table 2:** Mucosal Secretor Status among the Study Participants (n = 280).

	Saliva		Vagina		Cervix	
Secretor Phenotype	No.	%	No.	%	No.	%
**Secretors**	212	75.7	212	75.7	212	75.7
**Non-secretors**	68	24.3	68	24.3	68	24.3
**Total**	280		280		280	

### Secretor Status and Infection

Study participants were screened for HIV-1; 92 (32.9%) were HIV positive and 188 (67.1%) HIV negative. The proportion of secretors was significantly higher among women with HIV infection (77/92 = 83.7%) than among the HIV un-infected women (135/188 = 71.8%) (p = 0.029; OR, 1.647; 95% CI, 1.018–2.664). In addition all study participants underwent a detailed gynecological examination and samples were screened for *Bacterial vaginosis* (BV), *Neisseria gonorrhea* (GC) and *Trichomonas vaginalis* (TV) as indicated. For all sexually transmitted infection (STI) cases that tested positive, the secretors were a majority however; there was no significant difference in the incidence of infection between secretors and non-secretors when comparing those who tested positive for BV (p = 0.335; OR, 1.079; 95% CI, 0.926–1.257), GC (p = 0.930; OR, 1.021; 95% CI, 0.642–1.624) (Table 3).

**Table 3:** Correlation between Secretor Status and Sexually Transmitted Infection Rates among the Study Participants (n = 280).

	Secretors	Non-secretors	p Value
**Human Immunodeficiency Virus (n = 280)**			
Positive	77 (83.7%)	15 (16.3%)	p = 0.029
Negative	135 (71.8%)	53 (28.2%)	
***Trichomonas vaginalis* (n = 166)**			
Positive	11 (100%)	0 (0%)	-
Negative	123 (79.4%)	32 (20.6%)	
***Neisseria gonorrhea* (n = 167)**			
Positive	56 (81.2%)	13 (18.8%)	p = 0.930
Negative	79 (80.6%)	19 (19.4%)	
***Bacterial vaginalis* (n = 174)**			
Positive	64 (82.1%)	14 (17.9%)	p = 0.335
Negative	73 (76.0%)	23 (24.0%)	

### Blood Group Phenotypes and HIV Infection Rates

The incidence of HIV infection among the secretors was further stratified based on ABO blood groups. The incidence of HIV infection was the highest among blood group A secretors and when compared to blood group O, this difference was significant (p = 0.028; OR, 1.556; 95% CI, 1.061–2.280), blood group B, of modest significance (p = 0.066; OR, 1.625; 95% CI, 0.944–2.799) and blood group AB, not significant (p = 0.355; OR, 1.5; 95% CI, 0.573–3.930) as shown in Table 4.

**Table 4:** Comparison of Incidence of HIV Infections between the four ABO blood group phenotypes.

ABO Secretors	HIV Incidence (%)	Significance
**A** versus **O**	50 versus 32.1	p = 0.028
**A** versus **B**	50 versus 30.8	p = 0.066
**A** versus **AB**	50 versus 33.3	p = 0.355

## Discussion

This study is the first report to date on the correlation between secretor status and sexually transmitted infections (STIs) in Kenya. Specifically, this study correlated mucosal ABO blood group antigen expression in saliva, vaginal and cervical secretions to the incidence of HIV, *Bacterial vaginosis*, *Trichmonas vaginalis* and *Neisseria gonorrhea* among female sex workers in Nairobi, Kenya. The study population, female sex workers and their clients, account for 14.1% of new infections in Kenya. In addition, the concentrated epidemics among this high-risk population are known to have a significant impact on the STI prevalence dynamics. This was therefore a highly relevant study population to address the research question. Secretor status has been associated to a number of infections. This suggests a person’s secretor status; conferred by the *Fucosyltransferase 2* (*FUT2*) gene may be associated with varying disease susceptibilities. In some cases, the ABH secretors are more susceptible to infection, and in other instances the contrary is observed.

STI rates in the study population were HIV (32.8%), *Neisseria gonorrhea* (41.3%), *Bacterial vaginosis* (44.83%) and *Trichomonas vaginalis* (6.6%). The findings of this study demonstrate an increased incidence of STI infections among ABH secretors, but this difference was only statistically significant for HIV infections. There have been few studies on STI infections and secretor status [17–19]. A study on the correlation between secretor status and *Neisseria gonorrhea* [18] found similar prevalence rates of 42.8% compared to the 44.8% in the present study, but the correlation between secretor status and GC infections was statistically insignificant. To the best of our knowledge there have been no studies on the correlating between secretor status and *Bacterial vaginosis* or *Trichomonas vaginalis*. The increased incidence of HIV infections among ABH secretors in the present study compares with an earlier study by Ali *et al*., [17] among Senegalese commercial sex workers. In the study, secretor status was determined based on genotyping of the *FUT2* gene and correlated to HIV. Although the study screened for sexually transmitted infections, the association reported was only for HIV, where the incidence of HIV infections was more frequent among secretors, similar to the present study. A second study, among Swedish blood donors by Kindberg *et al*., [19], also based on *FUT2* genotyping associated the non-secretor phenotype with slower HIV disease progression. Together with our findings, these data suggests the presence of the ABH blood group antigens in mucosal secretions may increase an individual’s susceptibility to sexually transmitted infections specifically HIV while the non-secretor phenotype, lack of expression of ABO antigens in mucosal secretions, may confer a certain degree of protection against infection.

Interestingly blood group A secretors were found to have an increased incidence of HIV infections in comparison to their blood group AB, B and O counterparts. This has not been reported before, however studies have reported the occurrence of HIV in blood group O. In India blood group O individuals were found to have a higher incidence of infection [20] and a second study in Nigeria [21], reported similar findings–increased HIV incidence among blood group O individuals. However, for both studies the differences were statistically insignificant. Questions have been raised on the physiological significance of blood group antigens, aside from their well-described role in blood transfusion and compatibility testing. In this case, the basis underlying the expression of blood group antigens in the genital mucosa and HIV infection are not well understood; however there are several hypotheses. Pathogens including viruses and bacteria have been shown to selectively bind to the A, B and H blood group antigens via a family of receptors called lectins, which bind specifically to carbohydrate moieties [11]. It is postulated that these pathogens may initiate infection via non-covalent binding to these mucosal cell surface carbohydrate-binding proteins (lectins) [13, 14]. In this way, expression of the blood group antigens on mucosal surfaces in ABH secretors may place the host at an increased risk of infection (by aiding in pathogen attachment). On the contrary, modification of cell surface carbohydrates at mucosal surfaces in ABH non-secretors may in other instances offer a protective role [15].

Furthermore, various pathogens including bacteria and viruses express blood group antigen-identical or cross-reactive molecules on their surfaces [22, 23]. These pathogens have been postulated as probable targets for the corresponding blood group antibodies. Early studies on viral glycosylation [24] supported this line of thought. HIV isolates cultured *in vitro* with peripheral blood mononuclear cells from donors of different ABO groups demonstrated neutralization by the corresponding ABO antibodies of specific cell isolates. Preece *et al*., [25] demonstrated that measles virus, when co-cultured in a system expressing the ABH glycosyltransferases *in vitro*, expressed the corresponding A or B epitopes, according to the enzymes expressed. While, Neil *et al*., [26] highlight that HIV-1 can incorporate ABO blood group antigens, both in an artificial trans-infection system, and when primary strains are propagated in human peripheral blood mononuclear cells. Further investigations demonstrated that the presence of these antigens sensitize the virus to the serum of ABO-matched individuals by the action of heat labile complement in conjunction with anti-AB antibodies.

It was presumed for decades that blood group antigens were exclusively limited to transfusion and compatibility medicine. It is now apparent; these moieties are of clinical significance and may contribute to the provision of first line of defense against pathogens. In this regard, there have been no studies to investigate any possible associations between secretor phenotypes and infections in Kenya. In conclusion, this study demonstrates there is a correlation between secretor status, and HIV. However, there is need to confirm the role ABH blood group antigens in the context of HIV infections based on the hypotheses discussed above. These carbohydrate moieties possibly enhance viral binding and viral penetration, leading to establishment of infection, which may be particularly important at the initial stages of viral uptake into cells of the female genital tract. This may provide additional insight into the development of new HIV preventive technologies.

## Supporting Information

S1 Checklist (PDF)
