# Supplementary material for: Mucosal Blood Group Antigen Expression Profiles and HIV Infections: A Study among Female Sex Workers in Kenya
Source: PLoS One. 2015 Jul 17;10(7):e0133049. doi: 10.1371/journal.pone.0133049 (PMC4505875; doi:10.1371/journal.pone.0133049)
Supplement: S1 Checklist — (PDF) [file pone.0133049.s001.pdf]

STROBE Statement—checklist of items that should be included in reports of observational studies

	Item No	Recommendation	Page No.	Relevant text from manuscript
**Title and abstract**	1	(*a*) Indicate the study's design with a commonly used term in the title or the abstract	3	Lines (50); This cross-sectional study recruited 280 female sex workers aged 18-65 years from the Pumwani Majengo cohort, Kenya.
		(*b*) Provide in the abstract an informative and balanced summary of what was done and what was found	3	Lines (39-71); Abstract
**Introduction**				
Background/rationale	2	Explain the scientific background and rationale for the investigation being reported	4 and 5	Introduction (Lines 73-125)
Objectives	3	State specific objectives, including any prespecified hypotheses	5	Lines (122-125); This study was designed to investigate correlations between secretor status and HIV infection among female sex workers in Kenya based on the hypothesis, ABH secretors are more susceptible to HIV infections.
**Methods**				
Study design	4	Present key elements of study design early in the paper	5	Lines (128-129); This was a cross-sectional study conducted from January - October, 2013
Setting	5	Describe the setting, locations, and relevant dates, including periods of recruitment, exposure, follow-up, and data collection	5	Lines (128-144); Materials and methods
Participants	6	(*a*) *Cohort study*—Give the eligibility criteria, and the sources and methods of selection of participants. Describe methods of follow-up<br>*Case-control study*—Give the eligibility criteria, and the sources and methods of case	5	Lines (135-139); Study participants: 280 female sex workers aged 18-65 years presenting to sex worker outreach

		ascertainment and control selection. Give the rationale for the choice of cases and controls<br>*Cross-sectional study*—Give the eligibility criteria, and the sources and methods of selection of participants		program clinics for routine screening were recruited consecutively for this study.
		(*b*) *Cohort study*—For matched studies, give matching criteria and number of exposed and unexposed<br>*Case-control study*—For matched studies, give matching criteria and the number of controls per case	N/A	This was a cross-sectional study
Variables	7	Clearly define all outcomes, exposures, predictors, potential confounders, and effect modifiers. Give diagnostic criteria, if applicable	5 and 6	Lines (138-140); The sample size was calculated using a standard formula (Hayes and Bennett, 1999) to compare the proportion of individuals with the outcome of interest (Secretor phenotype).<br>Lines (189-192); The association between HIV-1 and secretor status was independent of several potentially confounding variables including, age, nationality, contraceptive use, and for *Bacterial Vaginosis*, *Neisseria gonorrhea* and *Trichomonas vaginalis* infections.
Data sources/ measurement	8*	For each variable of interest, give sources of data and details of methods of assessment (measurement). Describe comparability of assessment methods if there is more than one group	8	Lines (161-176); Sample Processing; Described screening of secretor status, all infections and blood groups.
Bias	9	Describe any efforts to address potential sources of bias	6	Lines (184-186); The presence of confounding was determined by comparing the ORs obtained from logistic regression models before and after addition of the covariates being evaluated. Stratified logistic regression analyses were performed to assess the relationship between HIV infection and the secretor phenotype with female sex

				workers; comparing HIV infected and HIV un-infected.
Study size	10	Explain how the study size was arrived at	5	Lines (138-144); The sample size was calculated using a standard formula (Hayes and Bennett, 1999)
Quantitative variables	11	Explain how quantitative variables were handled in the analyses. If applicable, describe which groupings were chosen and why	6	Lines (178-182); Mantel-Haenszel χ^2 tests were performed to compare dichotomous or categorical factors, with odds ratios (ORs) used as measures of association. Frequency distribution of the dependent categorical variables was compared by the Chi-square test.
Statistical methods	12	(*a*) Describe all statistical methods, including those used to control for confounding	6	Lines (178-192); Statistical analysis
		(*b*) Describe any methods used to examine subgroups and interactions	N/A	There were no subgroups
		(*c*) Explain how missing data were addressed	N/A	There was no missing data
		(*d*) *Cohort study*—If applicable, explain how loss to follow-up was addressed<br>*Case-control study*—If applicable, explain how matching of cases and controls was addressed<br>*Cross-sectional study*—If applicable, describe analytical methods taking account of sampling strategy	5	Lines (135-137); Based on a consecutive sampling approach, 280 female sex workers aged 18-65 years presenting to sex worker outreach program clinics for routine screening were recruited consecutively for this study
		(*e*) Describe any sensitivity analyses	N/A	
Results				
Participants	13*	(a) Report numbers of individuals at each stage of study—eg numbers potentially eligible, examined for eligibility, confirmed eligible, included in the study, completing follow-up, and analysed	N/A	This was a cross-sectional study with only one stage
		(b) Give reasons for non-participation at each stage	N/A	
		(c) Consider use of a flow diagram	N/A	
Descriptive data	14*	(a) Give characteristics of study participants (eg demographic, clinical, social) and information on exposures and potential confounders	6	(Line 204); Table 1. Socio-demographics characteristics of female

				sex workers recruited from the Pumwani Majengo Sex Worker cohort in Nairobi, Kenya (n=280)
		(b) Indicate number of participants with missing data for each variable of interest	N/A	There was no missing data
		(c) *Cohort study*—Summarise follow-up time (eg, average and total amount)	N/A	This was a cross-sectional study
Outcome data	15*	*Cohort study*—Report numbers of outcome events or summary measures over time	N/A	This was a cross-sectional study
		Case-control study—Report numbers in each exposure category, or summary measures of exposure	N/A	This was a cross-sectional study
		Cross-sectional study—Report numbers of outcome events or summary measures	8	Line (213); Table 2: Mucosal Secretor Status among the Study Participants
Main results	16	(*a*) Give unadjusted estimates and, if applicable, confounder-adjusted estimates and their precision (eg, 95% confidence interval). Make clear which confounders were adjusted for and why they were included	8	Line (213); Table 2: Mucosal Secretor Status among the Study Participants<br>Line (228); Table 3. Correlation between Secretor Status and Sexually Transmitted Infection Rates among the Study Participants (n=280)<br>Table 4. Comparison of Incidence of HIV Infections between the four ABO blood group phenotypes.
		(*b*) Report category boundaries when continuous variables were categorized	N/A	There were no continuous variables.
		(*c*) If relevant, consider translating estimates of relative risk into absolute risk for a meaningful time period	N/A	There were no estimates of relative risk
Other analyses	17	Report other analyses done—eg analyses of subgroups and interactions, and sensitivity analyses	N/A	There were no subgroups
**Discussion**				
Key results	18	Summarise key results with reference to study objectives	9	Lines (243-322); Discussion
Limitations	19	Discuss limitations of the study, taking into account sources of potential bias or imprecision. Discuss both direction and magnitude of any potential bias	N/A	
Interpretation	20	Give a cautious overall interpretation of results considering objectives, limitations, multiplicity of analyses, results from similar studies, and other relevant evidence	9 and 10	Lines (243-322); Discussion

Generalisability	21	Discuss the generalisability (external validity) of the study results	10	Lines (311-322); It was presumed for decades that blood group antigens were exclusively limited to transfusion and compatibility medicine. It is now apparent; these moieties are of clinical significance and may contribute to the provision of first line of defense against pathogens. In this regard, there have been no studies to investigate any possible associations between secretor phenotypes and infections in Kenya. In conclusion, this study demonstrates there is a correlation between secretor status, and HIV. However, there is need to confirm the role ABH blood group antigens in the context of HIV infections based on the hypotheses discussed above. These carbohydrate moieties possibly enhance viral binding and viral penetration, leading to establishment of infection, which may be particularly important at the initial stages of viral uptake into cells of the female genital tract. This may provide additional insight into the development of new HIV preventive technologies.
**Other information**				
Funding	22	Give the source of funding and the role of the funders for the present study and, if applicable, for the original study on which the present article is based		Submitted along with the online application form

*Give information separately for cases and controls in case-control studies and, if applicable, for exposed and unexposed groups in cohort and cross-sectional studies.

Note: An Explanation and Elaboration article discusses each checklist item and gives methodological background and published examples of transparent reporting. The STROBE checklist is best used in conjunction with this article (freely available on the Web sites of PLoS Medicine at http://www.plosmedicine.org/, Annals of Internal Medicine at http://www.annals.org/, and Epidemiology at http://www.epidem.com/). Information on the STROBE Initiative is available at www.strobe-statement.org.
